# Mesothelioma of the Tunica Vaginalis Testis: Diagnostic and Therapeutic Management. A Comprehensive Review, 1982–2024

**DOI:** 10.3390/cancers16233956

**Published:** 2024-11-26

**Authors:** Simona Stella, Giovanni Luca Ceresoli, Barbara Dallari, Rosalba Barile, Fabio Maisenti, Sabrina Rugarli, Alessandro Marinaccio, Dario Consonni, Carolina Mensi

**Affiliations:** 1 Occupational Health Unit, Fondazione IRCCS Ca’ Granda Ospedale Maggiore Policlinico, 20122 Milan, Italy; simona.stella@policlinico.mi.it (S.S.); barbara.dallari@policlinico.mi.it (B.D.); fabio.maisenti@policlinico.mi.it (F.M.); sabrina.rugarli@gmail.com (S.R.); dario.consonni@policlinico.mi.it (D.C.); 2 Department of Medical Oncology, Humanitas Gavazzeni Clinic, 24125 Bergamo, Italy; giovanni.ceresoli@mc.humanitas.it; 3 Department of Oncology, Saronno Hospital—ASST Valle Olona, 21047 Saronno, Italy; rosalba.barile@asst-valleolona.it; 4 Department of Occupational and Environmental Medicine, Epidemiology and Hygiene, Italian Workers’ Compensation Authority, 00143 Rome, Italy; a.marinaccio@inail.it

**Keywords:** mesothelioma, tunica vaginalis testis, rare cancers, treatment

## Abstract

Background: Mesothelioma of the tunica vaginalis testis (MTVT) is an extremely rare and aggressive cancer. The diagnosis and management of MTVT is complex, and no standard treatment protocol is available. Methods: We conducted a systematic literature review from 1 January 1982 to 14 March 2024 using PubMed to collect all the available case reports and case series. A descriptive analysis of patient characteristics with clinical presentation, diagnostic work-up, therapeutic management, and past asbestos exposure was performed. Survival times of patients treated with different therapeutic approaches were evaluated. Results: Overall, 289 patients with MTVT were included in our analysis. The most common clinical presentations were scrotal/testicular swelling or mass (187 patients, 65%) and the presence of hydrocele (159, 55%). Imaging evaluation, mostly with ultrasonography or CT scan, was reported in two-thirds of cases. Radical surgery (216 patients, 75%) with orchiectomy and, in select cases, hemiscrotectomy and inguinal lymphadenectomy was the most frequent therapeutic approach. A minority of patients (49, 17%) received adjuvant therapy after surgery (radiotherapy, chemotherapy, or a combination of the two), with no evidence of survival improvement. Conclusions: No standard guidelines for MTVT are available so far. Radical surgery following accurate radiological staging should be the mainstay of treatment. The role of adjuvant treatments remains undefined. Due to its rarity, MTVT should be treated in referral centers, and patients’ data should be collected in a dedicated register in order to improve the knowledge of this exceedingly rare disease and establish optimal diagnostic and therapeutic management.

## Simple Summary

Mesothelioma of the tunica vaginalis testis (MTVT) is an exceedingly rare and aggressive cancer, mostly related to previous exposure to asbestos. Diagnosis is complex, and there is currently no standard treatment protocol. Radical surgery following accurate radiological staging is the mainstay of treatment. The effect of adjuvant therapies (including radiotherapy, chemotherapy, or the combination of the two) on disease progression is not clear yet. Given the rarity of MTVT, the purpose of this narrative review is to collect and analyze the reported cases evaluating diagnostic and therapeutic management. The aim of this work was to improve the knowledge of this disease and to suggest proper diagnostic and therapeutic strategies.

## 1. Introduction

Mesothelioma is an aggressive malignant cancer arising from the serosal membranes that line the body cavities, including the pleura, peritoneum, pericardium, and scrotum. Mesothelioma of the tunica vaginalis testis (MTVT) is extremely rare, accounting for less than 5% of all mesotheliomas [1]. In Italy, the mean standardized (with the world standard population as a reference) incidence rate of MTVT is 0.095 per million person-years [2]. Its highest incidence occurs in subjects in their 60s, with a median age at diagnosis between 60 and 70 years [3], but it may present at any age, even under 20 years [4,5,6].

Asbestos exposure is a recognized risk factor [2]. Trauma, recurrent hydrocele, and a prolonged inflammation state may also be correlated with the development of the disease [7,8]. The most frequent symptoms include scrotal enlargement with a usually painless mass and hydrocele. MTVT has particularly aggressive behavior, with a high potential of metastatic disease; the reported median survival is less than 3 years [2,9]. The prognosis is worse in older patients [1,10]; other factors negatively influencing survival are non-epithelioid histology, a higher tumor grade, a higher stage, and lymph node involvement [1]. In the series of cases reviewed by Nazemi et al. [1], median OS was not reached in patients with T1 disease, while it was 1.7 years for patients with T4 disease (*p* = 0.002). Tumors greater than or equal to 4 cm were associated with worse OS (*p* = 0.025). Biphasic mesotheliomas had a dismal prognosis compared with the epithelioid subtype (*p* = 0.039), with an OS of 1.5 years. Recurrences of MTVT affect mostly inguinal, pelvic, retroperitoneal, and supraclavicular lymph nodes. Distant metastases may occur in the lung, liver, and bones [11].

Managing MTVT is particularly complex; preoperative diagnosis is uncommon, and most cases are diagnosed during or after surgery. Given the low incidence of MTVT, treatment guidelines have not been developed. Nearly all patients undergo extended surgical removal of the cancer, mostly by radical orchiectomy. Adjuvant therapies, comprising radiotherapy, chemotherapy, or a combination of the two, have been delivered in a minority of the reported cases. Despite these treatments, MTVT is associated with a high rate of recurrence that generally occurs within the first two years after primary surgery [5]. Recurrences are treated with palliative systemic treatment. In a few patients, lymph node recurrence may be treated with lymph node dissection.

This paper aimed to review published cases to analyze the clinical management of MTVT, with the goal of proposing a reasonable diagnostic and therapeutic approach to this rare disease.

## 2. Materials and Methods

### 2.1. Data Acquisition and Search Strategy

A systematic literature review was performed using the following string: “((testicular or paratesticular) and (mesothelioma or malignant mesothelioma)) or ((malignant mesothelioma or mesothelioma) and (tunica vaginalis or tunica vaginalis testis or testicular tunica or spermatic cord))”. We searched articles published from 1 January 1982, to 14 March 2024, using PubMed.

Only case reports and case series written in English were included, excluding clinical trials. We excluded articles with diagnoses of “benign mesothelioma” and “mesothelioma of uncertain malignant potential”. Articles were screened by title and abstract. The full text of potentially eligible papers was reviewed.

### 2.2. Types of Outcome Measures Included and Data Extraction

Whenever possible, data were gathered at the single-patient level. We collected articles with data concerning patient characteristics, asbestos exposure, clinico-pathological features, primary disease treatments (including surgery and adjuvant therapies), the pattern of recurrence, and follow-up information.

### 2.3. Statistical Analysis

Patients’ characteristics (including age at diagnosis, clinical presentation, histology, and asbestos exposure), diagnostic workups, and cancer therapeutic management were described. The survival times of two treatment groups (surgery only and surgery plus any adjuvant treatment), were calculated and compared using the Wilcoxon (Mann–Whitney) test. Data management and statistical analyses were performed using Stata 18 (Stata Corp. 2023, College Station, TX, USA) [12].

## 3. Results

**Figure 1:**
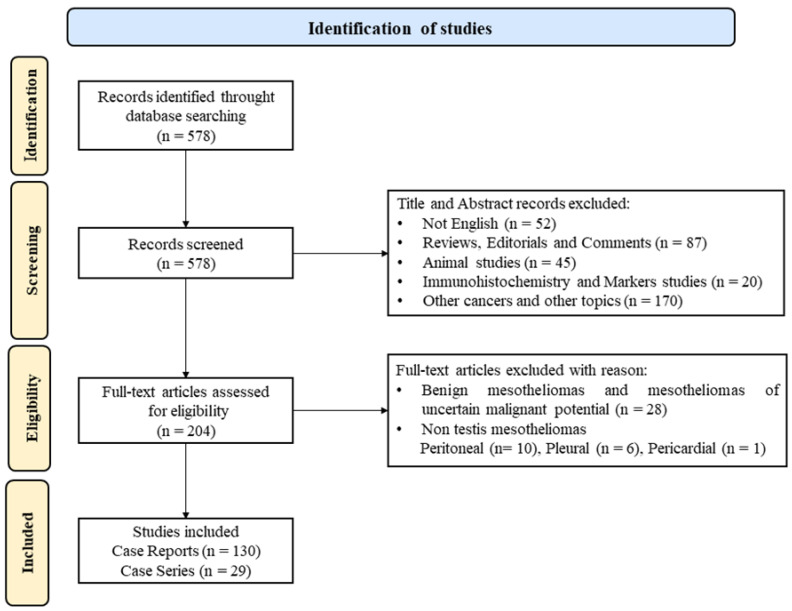
Flow chart of selection of studies on cases of mesothelioma of the tunica vaginalis testis.

We found and screened 578 articles (Figure 1); 419 were excluded by title or abstract. Overall, 159 papers (comprising 289 patients) were included in the final analysis (Supplementary Table S1) [3,5,7,8,9,10,13,14,15,16,17,18,19,20,21,22,23,24,25,26,27,28,29,30,31,32,33,34,35,36,37,38,39,40,41,42,43,44,45,46,47,48,49,50,51,52,53,54,55,56,57,58,59,60,61,62,63,64,65,66,67,68,69,70,71,72,73,74,75,76,77,78,79,80,81,82,83,84,85,86,87,88,89,90,91,92,93,94,95,96,97,98,99,100,101,102,103,104,105,106,107,108,109,110,111,112,113,114,115,116,117,118,119,120,121,122,123,124,125,126,127,128,129,130,131,132,133,134,135,136,137,138,139,140,141,142,143,144,145,146,147,148,149,150,151,152,153,154,155,156,157,158,159,160,161,162,163,164,165].

Table 1 summarizes the patient characteristics. The median age was 62 years (range: 6–93 years). Previous asbestos exposure was reported in 71 of 200 patients (36%) with available information, mostly occupational (52 out of 200, 26%). The most frequent clinical presentations were scrotal–testicular swelling and/or mass (161 cases, 56%), followed by hydrocele (159 cases, 55%) and pain (33 cases, 11%).

The patients’ diagnostic workups are summarized in Table 2. Pre-surgical imaging evaluation was reported in 195 of 289 cases (68%). Most patients underwent scrotum ultrasonography (138 patients, 48%) and computed tomography scans (CT scans, 114 patients, 40%). Cytological (including fine-needle aspiration) or histological examination (including surgical biopsies) was performed in 74 cases (26%). Tumor serum markers, including alpha-fetoprotein, beta-human chorionic gonadotropin, and lactate-dehydrogenase, were evaluated in 60 cases (21%) to exclude a germ cell tumor of the testis. The final histological diagnosis of MTVT with histotype subsets is reported in Table 3.

Table 4 shows the therapeutic management of the 289 reviewed MTVT cases. Almost all patients underwent surgery. Radical inguinal orchiectomy was performed in most cases (216, 75%); of these, 25 (9%) underwent more extended surgery with hemiscrotectomy and inguinal lymphadenectomy. Overall, 49 patients received adjuvant therapy, with either chemotherapy or radiotherapy (42, 15%) or both modalities (7, 2%). The radiotherapy dose ranged from 25 to 60.5 Gy. The majority of patients treated with chemotherapy received one (13 cases, 45%) or two (15 cases, 52%) agents; cisplatin (12 patients, 41%), pemetrexed (10 patients, 34%), doxorubicin (6 patients, 21%), and carboplatin (3 patients, 10%) were the most commonly used compounds. The median survival time was similar in patients treated with surgery and those treated with surgery plus adjuvant therapy.

A disease relapse was reported in 120 cases (42%), mostly with distant metastases (102 patients, 35%). Local recurrence occurred in 18 patients (6%).

## 4. Discussion

This study confirmed the lack of an established diagnostic and therapeutic approach for MTVT. The characteristics of the cases reviewed in our study were consistent with previously published data, except for a lower proportion of confirmed asbestos exposure. Most patients were treated with extended surgery, but a pre-surgical diagnosis of MTVT was uncommon. In patients for whom survival data were available, no prognostic benefit was observed with the addition of adjuvant treatments after surgery.

Asbestos exposure is the only well-established risk factor for MTVT [2]. Between 1993 and 2015, the Italian National Mesothelioma Registry (ReNaM) collected 80 MTVT cases. Occupational exposure to asbestos was found for 66% (45 out of 68 patients with available interview) and was associated with a higher risk of MTVT (odds ratio 3.42, 95% confidence interval 1.93–6.04). The higher percentage of occupational asbestos exposure in the ReNaM study (compared with 26% in our review) was expected. ReNaM is a nationwide population registry covering about 60 million people. It combines information collected in every Italian region by 21 Regional Operating Centers regarding all patients with mesothelioma (any site). ReNaM performs a thorough assessment of asbestos exposures; in particular, lifetime occupational and extra-occupational exposure to asbestos is collected through interviews with patients with mesothelioma (or their next-of-kin) using a standardized structured questionnaire, which is then evaluated by experienced personnel [109].

Preoperative diagnosis of MTVT is uncommon [141,164], and most cases are discovered incidentally during surgery [6,118,143]. Clinical presentation is unspecific; as previously reported [6,141], in this review the most frequent signs and symptoms were testicular/scrotal mass or swelling, hydrocele, and pain. Preoperative imaging studies, such as scrotal ultrasonography and abdominal CT scans, may reveal tunica surface irregularities or soft-tissue masses. Doppler ultrasound may show the vascular characteristics of MTVT. However, lesions can be small and difficult to see [10]. Contrast-enhanced ultrasound (CEUS) may represent a promising minimally invasive diagnostic tool for the characterization of testicular masses [166]. In pleural mesothelioma, CEUS was used to differentiate necrosis from vital tumor tissue, thereby decreasing the risk of false-negative results in bioptic samples. However, a specific contrast enhancement of mesothelioma in CEUS has not been described so far [167]. Serum tumor markers (lactate dehydrogenase, human chorionic gonadotropin, and alpha-fetoprotein) can be useful to rule out germ cell testicular cancer [105,165].

MTVT is characterized by epithelioid, biphasic, or sarcomatoid morphology with infiltrative growth into adjacent normal tissues. As for other localizations of mesothelioma, the epithelioid subtype is the most commonly reported subtype; in our study, 60% of cases had epithelioid morphology. The immunohistochemical MTVT profile is similar to that of pleural mesothelioma, including the expression of calretinin, WT1, and D2-40 [3,11]; MTAP and BAP-1 expression may be lost [168]. MTVT should be distinguished from extremely rare entities such as well-differentiated papillary mesothelial tumor (WDPMT) and mesothelioma of uncertain malignant potential (MUMP) of the tunica vaginalis, which are characterized by a benign clinical course if completely excised [111].

Due to the rarity of the disease, no standard guidelines for the treatment of MTVT are available [9,72,90,137,148,164]. Surgery is the mainstay of treatment [72]. In this review, 98% of patients were treated with tumor resection, mostly (75%) by orchiectomy. A minority of cases (nearly 10%) underwent hemiscrotectomy and/or inguinal lymph node dissection. The role of a more extensive surgery in MTVT is difficult to ascertain. More extensive surgery with hemiscrotectomy and en bloc orchiectomy was suggested specifically in more advanced cases and in patients previously treated with hydrocelectomy and an incidental diagnosis of MTVT in the hydrocelectomy specimen because of the risk of tumor seeding.

Some authors propose hemiscrotectomy with wide resection margins as an adjuvant surgical treatment [111]; in cases of limited lymph node involvement, lymph node dissection may be considered [6,111]. Faraj et al. published a small case series (six patients) treated with robot-assisted retroperitoneal lymph node dissection, claiming some diagnostic and therapeutic benefits with this procedure [150]. Follow-up monitoring of lymph node involvement with CT and/or MRI is advisable [111,134]. Managing patients with MTVT is challenging, not only because of the diagnostic and therapeutic complexity. The psychological distress of patients who are diagnosed with this cancer should not be underestimated. Patients with symptoms such as testicular swelling or testicular mass feel embarrassment [160]; radical surgery is a highly invasive procedure and may be accepted with difficulty or refused due to its consequences, as reported in several cases [63,115].

Adjuvant treatments after surgery (radiotherapy, chemotherapy, or the combination of the two) have been evaluated in a minority of patients with MTVT, with the aim of preventing metastatic spread and local recurrence [10]. Platinum-based chemotherapy, namely the combination of cisplatin and pemetrexed, was administered with apparent survival benefit [10]. In our review, 49 surgically treated patients (17%) received any adjuvant therapy (radiotherapy, platinum-based chemotherapy, or—in a few cases—both), with no significant improvement in survival compared with 233 patients (83%) treated with surgery alone. All the papers with available data were included in the survival analysis. Due to the limited number of cases receiving adjuvant treatment, no test of heterogeneity was performed. Therefore, the comparison between patients treated with surgery alone and those receiving any adjuvant treatment should be interpreted with caution.

Only a few case reports of patients with metastatic MTVT are available due to the rarity of this cancer. The benefit of systemic treatments is unclear, as these patients were mostly excluded from the main trials in mesothelioma, including the landmark Checkmate 743 study [169]. Among the reported cases, patients were treated with platinum-based chemotherapy, consistent with standard therapy for pleural mesothelioma. A partial response with first-line immunotherapy with nivolumab and ipilimumab in a case of MTVT with biphasic subtype histology was recently reported [9].

In conclusion, MTVT is a rare diagnosis, and the management strategies are not standardized in clinical guidelines. However, due to its aggressiveness, poor prognosis, and impact on quality of life, early detection and comprehensive treatment planning are crucial [3]. Therefore, diagnostic techniques such as scrotal ultrasonography (including Doppler ultrasound) and abdominal CT scans should be performed for every suspicious scrotal mass or in cases of recurrent hydrocele, especially if local pain is reported. If a malignancy is suspected, the diagnosis of MTVT should be confirmed pre-operatively by a fine-needle aspiration biopsy. A timely extensive surgical treatment is the standard of care; the role of adjuvant treatments is not defined. Patients should be ideally referred to centers with high expertise in this rare disease. Needless to say, psychological support can be important in improving the patient’s quality of life and adherence to the therapeutic plan. Recently, tools for assessing and managing the psychological distress in mesothelioma patients have been made available [170]. Finally, in Italy, epidemiological surveillance through cancer registries is mandatory to collect clinical details and asbestos exposure of MTVT cases [2]; moreover, aetiological and compensation issues have important impact on patients and their family’s quality of life. Only the accurate analysis of the diagnostic and therapeutic journey of a large number of MTVT cases will allow the establishment of evidence-based guidelines on this exceedingly rare but lethal disease.

## 5. Conclusions

In conclusion, MTVT has, on average, a very aggressive course and requires radical surgical treatment with a great psychological impact on patients. Finding the best treatment is crucial for patients’ physical and psychological well-being. Given the extreme rarity of the disease, no screening is indicated. However, the possible occurrence of MTVT in asbestos-exposed individuals should be considered; in this context, the activation of an MTVT national registry is fundamental.

## Figures and Tables

**Table 1:** Characteristics of patients with mesothelioma of the tunica vaginalis testis.

Variable	N	%
Total	289	100
Age, median (range)	62 (6–93)	
Side		
Left	122	42.2
Right	116	40.1
Bilateral	11	3.8
Not available	40	13.8
Asbestos exposure		
No	129	44.6
Yes	71	24.6
- Occupational	52	18.0
- Non-occupational	6	2.1
- Unspecified	13	4.5
Not available	89	30.8
Clinical presentation		
Hydrocele	159	55.0
Swelling/mass	187	64.7
- Scrotal/testicular	161	55.7
- Inguinal	19	6.6
- Both (scrotal/testicular and inguinal)	7	2.4
Pain	33	11.4
Other clinical presentations ^a^	24	8.3
Not available	14	4.8
Disease recurrence		
No	165	57.1
Local recurrence	18	6.2
Distant metastasis ^b^	102	35.3
Not reported	4	1.4

^a^ Including inguino-scrotal hernia, abdominal mass, edema of the penis, epididymitis, hematocele, spermatocele, cord torsion, chylous ascites, bilateral scrotoliths, swelling of the leg, and occasional diagnosis during herniorraphy and trauma. ^b^ Including lymph node recurrences.

**Table 2:** Diagnostic workup of mesothelioma of the tunica vaginalis testis.

Variable	N	%
Total	289	100
Imaging	195	67.5
Ultrasonography	138	47.8
Contrast-enhanced computed tomography scan	114	39.5
Positron emission tomography	16	5.5
Magnetic resonance imaging	13	4.5
Chest X-rays	29	10.0
Color Doppler ultrasound	14	4.8
Other ^a^	18	6.2
Pre-surgical cyto-histological diagnosis		
Cytological examination ^b^	46	15.9
Biopsy ^c^	28	9.7
Tumor markers ^d^	60	20.8

^a^ Including urogram, lymphangiography, and transillumination test. ^b^ Including fine-needle aspiration biopsy (FNAB) in nine cases. ^c^ Including surgical biopsy in 13 cases. ^d^ Including alpha-fetoprotein, beta-human chorionic gonadotropin, and lactate-dehydrogenase.

**Table 3:** Histological diagnosis of mesothelioma of the tunica vaginalis testis (ICD-O-3 code).

Variable	N	%
Total	289	100
Histotype		
Unspecified (90503)	46	15.9
Fibrous or sarcomatoid (90513)	4	1.4
Epithelioid (90523)	174	60.2
Biphasic (90533)	65	22.5

ICD-O-3, International Classification of Diseases for Oncology, 3rd Edition.

**Table 4:** Therapeutic management and survival of patients with mesothelioma of the tunica vaginalis testis.

Variable	N	%
Total	289	100
Surgery (any)	282	97.6
Orchiectomy	191	66.1
Orchiectomy + hemiscrotectomy	16	5.5
Orchiectomy + hemiscrotectomy + inguinal lymphadenectomy	9	3.1
Palliative surgery ^a^	66	22.8
Non-surgical therapy (any)		
Radiotherapy	27	9.3
Chemotherapy	29	10.0
**Survival according to treatment group ^b^**	**N (%)**	**Overall median survival (range) (months) ^c^**
Surgery only	233 (83)	24 (33–180)
Surgery plus adjuvant treatment (any)	49 (17)	24 (41–189)

^a^ Including hydrocelectomy in 32 cases (11.3%), herniorraphy, and tumorectomy with excision of masses/nodules. ^b^ Three patients did not receive surgery (two of these were in an advanced stage and one received palliative treatment); one patient refused surgery; one patient received only chemotherapy with cisplatin and doxorubicin. For two patients, the data were not available. ^c^ Survival data were available in 176 of 233 patients treated with surgery alone and in 43 of 49 patients receiving any adjuvant treatment. *p*-Value from Wilcoxon (Mann–Whitney) test: 0.59.

## Data Availability

No new data were created or analyzed in this study.

## Supplementary Materials

The following supporting information can be downloaded at: https://www.mdpi.com/article/10.3390/cancers16233956/s1, Table S1: Articles included in the review.
