# Supplementary material for: Mesothelioma of the Tunica Vaginalis Testis: Diagnostic and Therapeutic Management. A Comprehensive Review, 1982–2024
Source: Cancers (Basel). 2024 Nov 26;16(23):3956. doi: 10.3390/cancers16233956 (PMC11639812; doi:10.3390/cancers16233956)
Supplement: Supplementary file 1 [file cancers-16-03956-s001.zip › cancers-3306343-supplementary.pdf]

Supplementary Table S1. Articles included in the review.

N	Year	First author	Title	Reference	No. cases
1	1982	Chen K.T.	Malignant mesothelioma of tunica vaginalis testis.	Urol. 1982, 20, 316-319.	1
2	1982	Hollands M.J.	Malignant mesothelioma of the tunica vaginalis testis.	Eur Urol. 1982, 8, 121-2.	1
3	1982	Japko L.	Malignant mesothelioma of the tunica vaginalis testis: report of first case with preoperative diagnosis.	Cancer. 1982, 49, 119-127.	1
4	1982	Slaysman M.L.	Mesothelioma of the male genital tract in a patient with Maffucci's syndrome.	South Med J. 1982, 75, 1007-1010.	1
5	1983	Mc Donald R.E.	Paratesticular mesotheliomas.	J Urol. 1983, 130, 360- 361.	2
6	1983	van der Rhee H.J.	Cutaneous manifestations of malignant mesothelioma of the tunica vaginalis testis.	J Cutan Pathol. 1983, 10, 213-216.	1
7	1983	Zidar B.L.	Treatment of six cases of mesothelioma with doxorubicin and cisplatin.	Cancer. 1983, 52, 1788-1791.	1
8	1984	Yamanashi T.	Malignant mesothelioma of the tunica vaginalis testis	Eur Urol. 1984, 10, 207-209.	1
9	1985	Ehya H.	Cytology of mesothelioma of the tunica vaginalis metastatic to the lung.	Acta Cytol. 1985, 29, 79-84.	1
10	1985	Vakalikos I.	Malignant mesothelioma of testicular tunica.	J Surg Oncol. 1985, 29, 264.	1
11	1986	Karunaharan T.	Malignant mesothelioma of the tunica vaginalis in an asbestos worker.	J R Coll Surg Edinb. 1986, 31, 253-254.	1
12	1987	Cartwright L.E.	Malignant papillary mesothelioma of the tunica vaginalis testes: cutaneous metastases showing pagetoid epidermal invasion.	J Am Acad Dermatol. 1987, 17, 887-890.	1
13	1987	Fitzmaurice H.	Malignant mesothelioma of the tunica vaginalis testis.	Br J Urol. 1987, 60, 184.	1
14	1988	Prescott S.	Malignant mesothelioma of the tunica vaginalis testis: a case report.	J Urol. 1988, 140, 623-624.	1
15	1988	Velasco A.L.	Paratesticular malignant mesothelioma associated with abdominoscrotal hydrocele.	J Pediatr Surg. 1988, 23, 1065-1067.	1
16	1989	Grove A.	Mesotheliomas of the tunica vaginalis testis and hernial sacs.	Arch A Pathol Anat Histopathol. 1989, 415, 283-292.	3
17	1989	Tyagi G.	Malignant mesothelioma of tunica vaginalis testis.	Urol. 1989, 34, 102-104.	1
18	1990	Carp N.Z.	Malignant mesothelioma of the tunica vaginalis testis.	J Urol. 1990, 144, 1475-1478.	1
19	1990	Kamiya M.	Malignant mesothelioma of the tunica vaginalis.	Pathol Res Pract. 1990, 186, 680-686.	1
20	1990	Smith J.J.	Retroperitoneal lymph node dissection in malignant mesothelioma of tunica vaginalis testis	J Urol. 1990, 144,1242-1243.	1
21	1991	Kuwabara H.	Malignant mesothelioma of the tunica vaginalis testis. Report of a case and review of the literature	Acta Pathol Jpn. 1991, 41, 857-863.	1
22	1992	Adler B.D.	Malignant mesothelioma of the tunica vaginalis testis.	Australas Radiol. 1992, 36, 29-30.	1
23	1992	Fields J.M.	Case report: ultrasound appearances of a malignant mesothelioma of the tunica vaginalis testis.	Clin Radiol. 1992, 46, 128-30.	1
24	1992	Noble J.G.	Mesothelioma of the tunica vaginalis of the testis.	Br J Urol. 1992, 70, 328-329.	1
25	1992	Pfiter M.	Sonographic appearance of malignant mesothelioma of the tunica vaginalis testis in a child.	J Clin Ultrasound. 1992, 29, 129-131.	1
26	1992	Serio G.	Malignant mesothelioma of the testicular tunica vaginalis.	Eur Urol. 1992, 21, 174-176.	1
27	1994	Reynard J.M.	Malignant mesothelioma of the tunica vaginalis testis.	Br J Urol. 1994, 74, 389-390.	1
28	1994	Saw K.C.	Biphasic malignant mesothelioma of the tunica vaginalis testis.	Br J Urol. 1994, 74, 381-382.	1
29	1994	Wenger M.C.	Thymic rebound in a patient with scrotal mesothelioma.	J Thorac Imaging. 1994, 9, 145-147.	1

30	1995	Amin R.	Case report: malignant mesothelioma of the tunica vaginalis testis--an indolent course.	Br J Radiol. 1995, 68, 1025-1027.	1
31	1995	Eden G.C.	Malignant mesothelioma of the tunica vaginalis.	J Urol. 1995, 153, 1053-1054.	2
32	1995	Huncharek M.	Mesothelioma of the tunica vaginalis testis with possible occupational asbestos exposure.	Br J Urol. 1995, 75, 679-680.	1
33	1995	Jones M.A.	Malignant mesothelioma of the tunica vaginalis. A clinicopathologic analysis of 11 cases with review of the literature.	Am J Surg Pathol. 1995, 19, 815-825.	11
34	1995	Joseph A.K.	Purpuric nodules along a surgical scar. Metastatic malignant mesothelioma of the tunica vaginalis testis.	Arch Dermatol. 1995, 131, 484-5, 487-8.	1
35	1995	Lopez J.I.	Combined therapy in a case of malignant mesothelioma of the tunica vaginalis testis	Scand J Urol Nephrol. 1995, 29, 361-4.	1
36	1995	Tobioka H.	Multicystic mesothelioma of the spermatic cord.	Histopathol. 1995, 27, 479-481.	1
37	1995	Umewaka T.	Treatment of mesothelioma of the tunica vaginalis testis.	Urol Int. 1995, 55, 215-217.	1
38	1996	Ahmed M.	Malignant mesothelioma of the tunica vaginalis testis diagnosed by aspiration cytology--a case report with review of literature.	Int Urol Nephrol. 1996, 28, 793-796.	1
39	1996	Ascoli V.	Concomitant malignant mesothelioma of the pleura, peritoneum, and tunica vaginalis testis.	Diagn Cytopathol. 1996, 14, 243-248.	1
40	1996	Mathew B.S.	Case report: malignant mesothelioma of tunica vaginalis testis presenting with spinal metastasis--report of two cases.	Br J Radiol. 1996, 69, 1067-1068.	2
41	1997	Agapitos E.	Malignant mesothelioma of the tunica vaginalis testis: an immunohistochemical and ultrastructural study of two cases.	Br J Urol. 1997, 80, 345-346.	2
42	1997	Berti E.	Primary malignant mesothelioma of the tunica vaginalis of the testis. Immunohistochemistry and electron microscopy.	Pathol. 1997, 29, 96-99.	1
43	1997	Khan M.A.	Mesothelioma of tunica vaginalis testis in a child.	J Urol. 1997, 158, 198-199.	1
44	1998	Gupta S.C.	Pre-operative diagnosis of malignant mesothelioma of tunica vaginalis testis by hydrocele fluid cytology.	Eur J Surg Oncol. 1998, 24, 153-154.	1
45	1998	Lee M.	Mesothelioma of the tunica vaginalis--beware of the malignant hydrocoele.	Ir Med J. 1998, 91, 64-65.	2
46	1998	Plas E.	Malignant mesothelioma of the tunica vaginalis testis: review of the literature and assessment of prognostic parameters.	Cancer. 1998, 83, 2437-2446.	1
47	1999	Gupta N.P.	Malignant mesothelioma of the tunica vaginalis testis: a report of two cases and review of literature.	J Surg Oncol. 1999, 70, 251-254.	2
48	1999	Harmse J.L.	Malignant mesothelioma of the tunica vaginalis: a case with an unusually indolent course following radical orchidectomy and radiotherapy.	Br J Radiol. 1999, 72, 502-504.	1
49	1999	Kanazawa S.	Malignant mesothelioma of the tunica vaginalis testis: report of a case.	Surg Today. 1999, 29, 1106-1110.	1
50	2000	Attanoos R.L.	Primary malignant gonadal mesotheliomas and asbestos	Histopathol. 2000, 37, 150-159.	3
51	2000	Fujisaki M.	Case of mesothelioma of the tunica vaginalis testis with characteristic findings on ultrasonography and magnetic resonance imaging.	Int J Urol. 2000, 7, 427-430.	1
52	2000	Leiber C.	Tumour of the spermatic cord: an unusual primary manifestation of an epithelial mesothelioma of the peritoneum with patent processus vaginalis.	BJU Int. 2000, 86, 142-143.	1

53	2000	Poggi A.	A case of mesothelioma of the tunica vaginalis testis, with involvement of the pleura and peritoneum.	Tumori. 2000, 86, 256-257.	1
54	2001	Gürdal M.	Malignant mesothelioma of tunica vaginalis testis associated with long-lasting hydrocele: could hydrocele be an etiological factor?	Int Urol Nephrol. 2001, 32, 687-689.	1
55	2001	Sebbag G.	Malignant mesothelioma of the male genital tract: Report of two cases.	Urol Oncol Orig Investig. 2001, 6, 261-264.	2
56	2001	Wolanske K.	Malignant mesothelioma of the tunica vaginalis testis: atypical sonographic appearance.	J Ultrasound Med. 2001, 20, 69-72.	1
57	2002	Abe K.	Malignant mesothelioma of testicular tunica vaginalis.	Int J Urol. 2002, 9, 602-603.	1
58	2002	Bruno C.	Diagnosis of malignant mesothelioma of the tunica vaginalis testis by ultrasound-guided fine-needle aspiration.	J Clin Ultrasound. 2002, 30, 181-183.	1
59	2002	Iczkowski K.A.	Malignant mesothelioma of tunica vaginalis testis: a fatal case with liver metastasis.	J Urol. 2002, 167, 645-646.	1
60	2003	Black P.C.	Extensive palliative surgery for advanced mesothelioma of the tunica vaginalis.	Urol. 2003, 62, 748.	1
61	2003	García de Jalón A.	Malignant mesothelioma of the tunica vaginalis. Report of a case without risk factors and review of the literature.	Int Urol Nephrol. 2003, 35, 59-62.	1
62	2004	Mak C.W.	Malignant mesothelioma of the tunica vaginalis testis.	Br J Radiol. 2004, 77, 780-781.	1
63	2004	Pelzer A.	Synchronous bilateral malignant mesothelioma of tunica vaginalis testis: early diagnosis.	Urol. 2004, 64, 1031.	1
64	2004	Sawada K.	Multicystic malignant mesothelioma of the tunica vaginalis with an unusually indolent clinical course.	Hinyokika Kiyo. 2004, 50, 511-513.	1
65	2004	Shimada S.	Malignant mesothelioma of the tunica vaginalis testis: a case with a predominant sarcomatous component.	Pathol Int. 2004, 54, 930-934.	1
66	2005	Gorini G.	Mesothelioma of the tunica vaginalis testis: report of 2 cases with asbestos occupational exposure.	Int J Surg Pathol. 2005, 13, 211-214.	2
67	2005	Spiess P.E.	Malignant mesothelioma of the tunica vaginalis.	Urol. 2005, 66, 397-401.	5
68	2005	Torbati P.M.	Malignant mesothelioma of the spermatic cord: case report and review of the literature.	Urol J. 2005, 2, 115-117.	1
69	2005	Wang M.T.	Malignant mesothelioma of the tunica vaginalis testis: unusual sonographic appearance.	J Clin Ultrasound. 2005, 33, 418-420.	1
70	2006	Schure P.J.	Mesothelioma of the tunica vaginalis testis: a rare malignancy mimicking more common inguino-scrotal masses.	J Surg Oncol. 2006, 94, 161-164.	3
71	2006	van Apeldoorn M.J.	Mesothelioma of the tunica vaginalis complicated by chyluria.	J Clin Oncol. 2006, 24, 5329-5330.	1
72	2006	Winstanley A.M.	The immunohistochemical profile of malignant mesotheliomas of the tunica vaginalis: a study of 20 cases.	Am J Surg Pathol. 2006, 30, 1-6.	18
73	2007	Al-Qahtani M.	Malignant mesothelioma of the tunica vaginalis.	Can J Urol. 2007, 14, 3514-3517.	1
74	2007	Guney N.	Malignant mesothelioma of the tunica vaginalis testis: a case report and review of the literature.	Med Oncol. 2007, 24, 449-452.	1
75	2007	Liguori G.	Inguinal recurrence of malignant mesothelioma of the tunica vaginalis: one case report with delayed recurrence and review of the literature.	Asian J Androl. 2007, 9, 859-860.	1
76	2008	Boyum J.	Malignant mesothelioma of the tunica vaginalis testis: a case illustrating Doppler color flow imaging and its potential for preoperative diagnosis.	J Ultrasound Med. 2008 , 27, 1249-1255.	1
77	2008	Candura S.M.	Malignant mesothelioma of the tunica vaginalis testis in a petrochemical worker exposed to asbestos.	Anticancer Res. 2008, 28, 1365-1368.	1

78	2008	Goel A.	Malignant mesothelioma of the tunica vaginalis of the testis without exposure to asbestos.	Cases J. 2008, 1, 310.	1
79	2008	Ikegami Y.	Malignant mesothelioma of the tunica vaginalis testis related to recent asbestos.	Int J Urol. 2008, 15, 560-561.	1
80	2008	Mathur S.R.	Malignant mesothelioma of tunica vaginalis: a report of 2 cases with preoperative cytologic diagnosis.	Acta Cytol. 2008, 52, 740-743.	2
81	2009	Al-Salam S.	Expression of Wilms tumor-1 protein and CD 138 in malignant mesothelioma of the tunica vaginalis.	Pathol Res Pract. 2009, 205, 797-800.	1
82	2009	Bacchetta J.	Mesothelioma of the testis and nephrotic syndrome: a case report.	J Med Case Rep. 2009, 3, 7248.	1
83	2009	Chen J.L.	Malignant mesothelioma of the tunica vaginalis testis: a case report and literature review.	Kaohsiung J Med Sci. 2009, 25, 77-81.	1
84	2009	De Lima G.R.	A rare case of malignant hydrocele in a young patient	J Pediatr Urol. 2009, 5, 243-245.	1
85	2010	Aggarwal P.	Preoperative diagnosis of malignant mesothelioma of the tunica vaginalis using Doppler ultrasound.	Urol. 2010, 75, 251-252.	1
86	2010	Bisceglia M.	Report of a case with comprehensive review of literature.	Adv Anat Pathol. 2010, 17, 53-70.	1
87	2010	Klaassen Z.	Malignant mesothelioma of the tunica vaginalis testis: A rare, enigmatic tumor.	Urotoday Int J. 2010, 3	1
88	2011	Bass L.	Multiple primary cancers including mesothelioma of the tunica vaginalis: case report and literature review with primary care focus.	J Am Osteopath Assoc. 2011, 111, 483-486.	1
89	2011	Grey Venyo A.	Malignant mesothelioma of tunica vaginalis testis complicating a hydrocele.	WebmedCentral Urology. 2011, 2, WMC002309.	1
90	2011	Gupta R.	Fine needle aspiration cytology in malignant mesothelioma of the tunica vaginalis testis.	Cytopathol. 2011, 22, 66-68.	1
91	2011	Park Y.J.	Malignant mesothelioma of the spermatic cord.	Korean J Urol. 2011, 52, 225-229.	1
92	2012	Abdelrahman M.	Malignant mesothelioma of the tunica vaginalis.	J Surg Case Rep. 2012, 2012, 2.	1
93	2012	Ahmed S.	Epithelioid malignant mesothelioma of tunica vaginalis with deciduoid features: an unusual malignancy clinically masquerading an inguinal hernia.	Indian J Pathol Microbiol. 2012, 55, 89-91.	1
94	2012	Doo S.W.	Radiologic findings of mesothelioma at the tunica vaginalis.	Urol. 2012, 80, e3-5.	1
95	2012	Esen T.	Malignant mesothelioma of the tunica vaginalis: presenting with intermittent scrotal pain and hydrocele.	Case Rep Med. 2012, 2012:189170.	1
96	2012	Hai B.	Diagnosis and prognosis of malignant mesothelioma of the tunica vaginalis testis.	Can Urol Assoc J. 2012, 6, E238-E241.	6
97	2012	Mensi C .	Mesothelioma of tunica vaginalis testis and asbestos exposure.	BJU Int. 2012, 110, 533-537.	13
98	2012	Mrinakova B.	Paratesticular mesothelioma in young age. Case report.	Klin Onkol. 2012, 25, 290-293.	1
99	2012	Priester P.	Cutaneous recurrence of malignant mesothelioma of the tunica vaginalis testis: a rare case report.	Onkologie. 2012, 35, 46-48.	1
100	2012	Vijayan S.	Primary and Secondary Mesothelioma of the Tunica Vaginalis: a comparative case study.	BJU Int. 2012.	1
101	2012	Yen C.H.	Malignant mesothelioma of the tunica vaginalis testis: a malignancy associated with recurrent epididymitis?	World J Surg Oncol. 2012, 10, 238.	1
102	2013	Busto Martin L.	Mesothelioma of the tunica vaginalis. Case report.	Arch Esp Urol. 2013, 66, 384-388.	1
103	2013	Gkentzis A.	An unusual case of paratesticular mesothelioma on the site of previously excised epididymal adenomatoid tumour	Int J Surg Case Rep. 2013, 4, 460-462.	1
104	2013	Meng X.	Malignant mesotheliomas in spermatic cords: reports of two cases and a brief review of literature.	Rare Tumors. 2013, 5, e4.	2

105	2013	Rajan V.	Paratesticular malignant mesothelioma - a rare case presentation.	Indian J Surg. 2013, 75, 174-176.	1
106	2013	Weng C.H.	Incidentally discovered malignant mesothelioma of the tunica vaginalis.	J Formos Med Assoc. 2013, 112, 57-58.	1
107	2014	De Fonseca L.G.	Malignant paratesticular mesothelioma.	Autops Case Rep. 2014, 4, 45-51.	1
108	2014	Hsu L.N.	Paratesticular malignant mesothelioma in a patient exposed to asbestos for more than 50 years.	Kaohsiung J Med Sci. 2014, 30, 537-538.	1
109	2014	Stradella A.	Malignant mesothelioma of tunica vaginalis.	Actas Urol Esp. 2014, 38, 68-69.	1
110	2014	Yang L.H.	Mesothelioma of the tunica vaginalis testis with prominent adenomatoid features: a case report.	Int J Clin Exp Pathol. 2014, 7, 7082-7087.	1
111	2015	Akin Y.	Malignant mesothelioma of tunica vaginalis: an extremely rare case presenting without risk factors.	Singapore Med J. 2015, 56, e53-e55.	1
112	2015	Alesawi A.M.	Malignant mesothelioma of the tunica vaginalis testis: comprehensive review of literature and case report.	J Clin Urol. 2015, 147-152.	1
113	2015	Bandyopadhyay A.	Preoperative cytological diagnosis of malignant mesothelioma of tunica vaginalis.	Diagn Cytopathol. 2015, 43, 850-854.	1
114	2015	Jankovichova T.	Extremely rare tumour--malignant mesothelioma of tunica vaginalis testis.	Bratisl Lek Listy. 2015, 116, 574-576.	1
115	2015	Segura-González M.	Malignant mesothelioma of the tunica vaginalis: a rare neoplasm--Case report and literature review	Clin Genitourin Cancer. 2015, 13, e401-e405.	1
116	2016	Ahmed Z.	Primary malignant mesothelioma of the spermatic cord.	BMJ Case Rep. 2016, 2016, bcr2016214602.	1
117	2016	Andresen E.D.	Malignant mesothelioma of the tunica vaginalis: a rare case report and description of multimodal treatment.	Can J Urol. 2016, 23, 8585-8589.	1
118	2016	Bertolotto M.	Imaging of mesothelioma of tunica vaginalis testis.	Eur Radiol. 2016, 26, 631-638.	10
119	2015	D'Antonio A.	Malignant mesothelioma of spermatic cord in an elderly man with a history of asbestos exposure.	Urol. 2016, 87, e1-e3.	1
120	2016	Hispan H.	Cutaneous metastases from malignant mesothelioma of the tunica vaginalis testis.	Am J Dermatopathol. 2016, 38, 222-225.	1
121	2016	Monton C.S.	Mesothelioma of the tunica vaginalis in a patient with giant hydrocele.	Radiol Bras. 2016, 49, 63-64.	1
122	2016	Mrinakova B.	Malignant mesothelioma of the tunica vaginalis testis. A clinicopathologic analysis of two cases with a review of the literature.	Klin Onkol. 2016 , 29, 369-374.	2
123	2016	Serio G.	Molecular changes of malignant mesothelioma in the testis and their impact on prognosis: analyses of two cases.	Int J Clin Exp Pathol. 2016, 9, 7658-7667.	1
124	2017	An J.Y .	Clinical features and outcomes of tunica vaginalis mesothelioma: A case series from the National Institutes of Health.	Clin Genitourin Cancer. 2017, 15, e871-e875.	7
125	2017	Arda E.	Malignant mesothelioma of tunica vaginalis testis: macroscopic and microscopic features of a very rare malignancy.	Cureus. 2017, 9, e1860.	1
126	2017	Maheshwari P.N.	Hydrocele with a surprise: Malignant mesothelioma of the tunica vaginalis - Case report and review of literature.	Urol Ann. 2017, 9, 110-112.	1
127	2017	Recabal P.	Malignant mesothelioma of the tunica vaginalis testis: Outcomes following surgical management beyond radical orchiectomy.	Urol. 2017, 107, 166-170.	15
128	2017	Shaikh A.A.	Bilateral malignant mesothelioma of tunica vaginalis A case report on rare presentation.	Urol Case Rep. 2017, 14, 53-55.	1
129	2017	Zhang N.	Malignant mesothelioma of the tunica vaginalis testis: A case report and literature review.	Mol Clin Oncol. 2017, 7, 1053-1056.	1

130	2018	Abello A.	A bilateral metachronous mesothelioma of the tunica vaginalis.	Urol. 2018, 120, e1-e2.	1
131	2018	Emile S.H.	Malignant mesothelioma of the tunica vaginalis: Incidental diagnosis of a rare condition.	Indian Journal of Surgery. 2018, 81, 80-82.	1
132	2018	Priyadarshi N.	Malignant mesothelioma of testis: A report of three cases and review of literature.	Med J of Dr. D.Y. Patil Vidyapeeth. 2018, 11, 60-62.	3
133	2018	Trenti E.	Malignant mesothelioma of tunica vaginalis testis: Report of a very rare case with review of the literature.	Arch Ital Urol Androl. 2018, 90, 212-214.	1
134	2018	Zhang S.	Genome evolution analysis of recurrent testicular malignant mesothelioma by whole-genome sequencing.	Cell Physiol Biochem. 2018, 45, 163-174.	1
135	2019	Baqui A.A.	Malignant mesothelioma of the tunica vaginalis testis-A malignancy associated with asbestos exposure and trauma: A case report and literature review.	J Investig Med High Impact Case Rep. 2019, 7, 2324709619827335.	1
136	2019	Brun C.	The necessity of a more aggressive initial surgical treatment in patients with mesothelioma of the testicular tunica vaginalis.	Ann Med Surg, 2019, 47, 57-60.	1
137	2019	Butnor K.J.	Mesothelioma of the tunica vaginalis testis.	Hum Pathol. 2019, 92, 48-58.	20
138	2019	Faraj K.S.	Role of robot-assisted retroperitoneal lymph node dissection in malignant mesothelioma of the tunica vaginalis: case series and review of the literature.	Can J Urol. 2019, 26, 9752-9757.	6
139	2019	Harris M.C.	Malignant mesothelioma of the tunica vaginalis: a atypical presentation of a rare scrotal tumour.	ANZ J Surg. 2019, 89, E562-E563.	1
140	2019	Punatar C.B.	Malignant mesothelioma of tunica vaginalis without any risk factors: An uncommon case.	J Cancer Res Ther. 2019, 15, S167-S169.	1
141	2019	White K.D.	An incidental finding of paratesticular malignant mesothelioma during operation for hydrocelectomy.	S D Med. 2019, 72, 532-534.	1
142	2020	Drevinskaite M.	Malignant mesothelioma of the tunica vaginalis testis: a rare case and review of literature.	BMC Cancer. 2020, 20, 162.	1
143	2020	Hannappel T.D.	Imaging appearance of cystic and solid mesothelioma of the tunica vaginalis.	Radiol Case Rep. 2020, 15, 809-811.	1
144	2020	Kazaz I.O.	Mesotelioma of TVT: a case report.	Indian J Pathol Microbiol. 2020, 63, 475-477.	1
145	2020	Kilitci A.	Sarcomatoid type of paratesticular malignant mesothelioma in a dry-cleaning worker exposed to asbestos and diagnostic value of WT-1.	P R Health Sci J. 2020, 39, 39-44.	1
146	2020	Kobayashi Y.	Mesothelioma in situ of the spermatic cord arising from a patent processus vaginalis: A case report.	Urol J. 2020, 17, 671-673.	1
147	2020	Singh I.	Malignant mesothelioma of the spermatic cord mimicking a benign inguinoscrotal swelling: case report with review of literature.	Afr J Urol. 2020, 26, 87.	1
148	2022	de Sá Barrêto Callou Peixoto M.	Malignant mesothelioma of the tunica vaginalis testis: A rare cause of hydrocele.	Urol Case Rep. 2022, 43, 102048.	1
149	2022	Matzuki R.	A case of simultaneous diagnosis of tunica vaginalis testis and pleural mesothelioma.	Respirol Case Rep. 2022, 10, e0937.	1
150	2022	Zafar R.	A diagnostic approach to paratesticular lesions with tubulopapillary architecture: a series of 16 serous borderline tumors/low-grade serous carcinoma and 14 well-differentiated papillary mesothelial tumors and mesothelioma.	Hum Pathol. 2022, 128, 31-47.	12
151	2023	Dytor T.	Malignant mesothelioma of the tunica vaginalis and epididymis.	Diagnostic Histopathology. 2023, 2:5.	1

152	2023	Janes W.C.I.	Malignant mesothelioma of the testes with retroperitoneal recurrence and resection in an 80-year-old male and review of the literature.	Case Rep Oncol. 2023, 16, 698-704.	1
153	2023	Karabacakoglu B.	Malignant mesothelioma of tunica vaginalis: Two extremely rare case reports.	Urologia. 2023, 90, 422-425.	2
154	2023	Mishra K.	A rare presentation of malignant mesothelioma of the tunica vaginalis managed with immunotherapy and review of the literature.	Clin Case Rep. 2023, 11, e7610.	1
155	2024	Behers B.M.	Malignant epithelioid mesothelioma of the tunica vaginalis testis presenting as hydrocele in a kidney transplant recipient.	Case Rep Urol. 2024, 2024, 9227764.	1
156	2024	Jiang Y.	A rare malignant mesothelioma of the tunica vaginalis testis: A case report	Oncol Lett. 2024, 27, 172.	1
157	2024	Kim K.H.	Patient with recurrent paratesticular malignant mesothelioma and multiple primary neoplasms.	JOMH. 2024. 20, 114-119.	1
158	2024	Nazar T.	Testicular mesothelioma disguised as hydrocele: a case report.	J Med Case Rep. 2024, 18, 114.	1
159	2024	Shaker N.	Malignant para-testicular mesothelioma: A rare presentation in the tunica vaginalis of an elderly male with no prior asbestos exposure.	Int J Surg Pathol. 2024, 32: 1117-1122.	1
